# Isoflavonoid-Rich *Flemingia macrophylla* Extract Attenuates UVB-Induced Skin Damage by Scavenging Reactive Oxygen Species and Inhibiting MAP Kinase and MMP Expression

**DOI:** 10.1155/2013/696879

**Published:** 2013-07-01

**Authors:** Hsiu-Mei Chiang, Hua-Hsien Chiu, Sue-Tsai Liao, Yen-Ting Chen, Hsien-Chang Chang, Kuo-Ching Wen

**Affiliations:** ^1^Department of Cosmeceutics, China Medical University, Taichung 404, Taiwan; ^2^Center for Biomedical Technology Research and Development, Fooyin University, Kaohsiung 831, Taiwan; ^3^Brion Research Institute of Taiwan, Taipei 231, Taiwan

## Abstract

In this study, we investigated the antioxidant activity and anti-photoaging properties of an extract of *Flemingia macrophylla*, a plant rich in isoflavonoid content. Pretreatment of fibroblasts with *Flemingia macrophylla* extract (FME) inhibited elastase activity, promoted the protein expression of type I procollagen, and attenuated the phosphorylation of mitogen-activated protein (MAP) kinase and the protein expression of matrix-metalloproteinase- (MMP-) 1, 3, and 9. The IC_50_ values were 2.1 **μ**g/mL for DPPH radical scavenging ability, 366.8 **μ**g/mL for superoxide anion scavenging ability, 178.9 **μ**g/mL for hydrogen peroxide scavenging ability, and 230.9 **μ**g/mL for hydroxyl radical scavenging ability. Also, exposure of erythrocytes to various concentrations of FME (50–500 **μ**g/mL) resulted in a dose- and time-dependent inhibition of AAPH-induced hemolysis. In human fibroblasts, FME at 10 **μ**g/mL was shown to be a potent scavenger of UV-induced reactive oxygen species (ROS). The antioxidant and anti-photoaging properties of FME make it an ideal anti-intrinsic aging and anti-photoaging agent.

## 1. Introduction

Overexposure to ultraviolet (UV) irradiation can result in edema, erythema, inflammation, hyperpigmentation, hyperplasia, and photoaging as well as DNA damage and mutations in skin [1]. Depletion of the ozone layer and changes in outdoor lifestyle have resulted in increased exposure of humans to UV irradiation. Photoaging due to chronic exposure to short wavelength UV light (UVB) is characterized by severe wrinkling, roughness, sagging, and pigmentary changes, such as solar lentigo and mottled pigmentation on exposed areas. UVB has been shown to stimulate the generation of reactive oxygen species (ROS), such as hydroxyl radical and H_2_O_2_, leading to oxidative stress and subsequently skin damage [2, 3]. Studies have demonstrated that UVB-induced oxidative stress causes cell structure damage and affects the repair and transcription of DNA as well as the synthesis of proteins [4, 5]. UVB-induced ROS can also initiate lipid peroxidation and produce lipid alkoxy radicals, which can have a photocarcinogenic effect due to the formation of DNA adducts.

UVB irradiation damages the antioxidant defense system, impairs signal transduction pathways in skin cells, and degrades extracellular matrix (ECM) proteins, including collagen, elastin, proteoglycans, and fibronectin [6, 7]. Furthermore, it has been demonstrated that ROS affects signal transduction pathways of growth factor receptors, cytokine receptors, mitogen-activated protein (MAP) kinases, and activator protein-1 (AP-1) and activates matrix metalloproteinases (MMPs), resulting in the degradation of ECM proteins and subsequent formation of coarse wrinkles and sagging skin [7]. Type I collagen, the major structural protein of skin connective tissue, is synthesized primarily by fibroblasts and is responsible for conferring strength and elasticity of skin [8]. MMPs are known to be overexpressed in human fibroblasts after UVB exposure and are, therefore, considered as key regulators in the photoaging process [9]. MMP-1 degrades collagen, gelatin, and proteoglycan link protein, and MMP-3 is involved in several MMP activation cascades including activation of MMP-1 [10]. Therefore, agents with the ability to scavenge ROS, elevate ECM protein levels, or inhibit the major collagen-degrading enzymes, such as MMPs, would prove useful in the development of effective antiaging agents.

The root and stems of *Flemingia macrophylla*, a polyphenol-rich member of the Fabaceae family of flowering plants, are used as a folk medicine in many Asian countries to treat rheumatic, inflammatory, and blood circulation disorders [11]. It has been reported that *Flemingia macrophylla* extract (FME) shows analgesic and anti-inflammatory activities in mice [12] as well as antioxidant and hepatoprotective effects in rats [13]. Furthermore, the flavonoids in *Flemingia macrophylla* have been demonstrated to inhibit A*β*-induced neurotoxicity [11]. *Flemingia macrophylla* is rich in polyphenols such as daidzin, daidzein, genistin, genistein, flemingin A, and flemingin D [11, 14]. Many isoflavonoids also have significant inhibitory effects on MMPs in dermal fibroblasts and chemoprotective effects against skin cancer. Genistin was reported to inhibit UV-induced DNA damage [15], and its aglycone, genistein, was shown to prevent skin damage in mice [16] and UVB-induced premature senescence in human fibroblasts [17]. In our previous study, we found that daidzein and genistein derived from Radix Puerariae stimulated hyaluronic acid production in normal human epidermal keratinocytes [18]. In this study, we investigated the antioxidant activities of FME and its effects on protein expression of MMPs, elastase, and type I procollagen in human dermal fibroblasts after exposure to UVB.

## 2. Materials and Methods

### 2.1. Preparation of *Flemingia macrophylla* Extract (FME)

Stems of *Flemingia macrophylla* were harvested in Pingtung County, Taiwan. The dried stems were ground and then extracted twice by boiling for 2 h in a 20-fold volume of water. The supernatant was filtered and then evaporated to dryness in a freeze dryer. The extract was stored at −20°C before use.

### 2.2. Chemicals

Acetonitrile, methanol, dimethyl sulfoxide (DMSO), phosphoric acid, gallic acid, quercetin, aluminum chloride hexahydrate (AlCl_3_), potassium acetate (CH_3_COOK), calcium chloride (CaCl_2_), propylene glycol (PG), DL-dithiothreitol, Folin-Ciocalteu reagent, 1,1-diphenyl-2-picrylhydrazyl radical (DPPH), 2,2′-azobis(2-methylpropionamidine) dihydrochloride (AAPH), FeCl_2_, 2-thio-barbituric acid (TBA), 2′,7′-dichlorofluorescin diacetate (DCFDA), 3-(2-pyridyl)-5,6-diphenyl-1,2,3-triazine-4′,4′′-disulfonic acid sodium salt (Ferrozine), butanol, pyridine, sodium nitrite, trichloroacetic acid (TCA), and deoxyribose were purchased from Sigma-Aldrich Chemicals (St. Louis, MO, USA). Fetal bovine serum (FBS), penicillin-streptomycin, trypsin-EDTA, and Dulbecco's Modified Eagle's Medium (DMEM) were purchased from Gibco, Invitrogen (Carlsbad, CA, USA). Bradford reagent was supplied by Bio-Rad Laboratories (CA, USA). Coomassie Blue R-250, dibasic sodium phosphate, Igepal CA-630, tris, and 3-(4,5-dimethylthiazol-2-yl)-2,5-diphenyltetrazolium bromide (MTT) were purchased from USB (Cleveland, OH, USA). Collagenase was purchased from Calbiochem, Merck (Darmstadt, Germany) and Fluorogenic Peptide Substrate I was purchased from R & D systems (Wiesbaden, Germany). Human foreskin fibroblasts were obtained from the Bioresource Collection and Research Center (Hsinchu, Taiwan).

### 2.3. Quantitation of FME

#### 2.3.1. Total Phenolic Content of FME

The total phenolic content of FME was determined by the Folin-Ciocalteu reaction as described previously [19, 20]. Briefly, a mixture of FME, Folin-Ciocalteu phenol reagent, and sodium carbonate was prepared. The mixture was then centrifuged and the absorbance of the supernatant was measured at a wavelength of 760 nm using a spectrophotometer (PerkinElmer Inc. USA). Gallic acid was used as the standard for the calibration curve and the phenolic contents were calibrated using a linear equation based on the calibration curve. The contents of phenolic compounds are expressed as *μ*g gallic acid equivalent (GAE)/mg *Flemingia macrophylla* stem dry weight.

#### 2.3.2. Quantitation of FME Using a High-Performance Liquid Chromatography-Diode Array Detector (HPLC-DAD) Method

The HPLC apparatus was equipped with a pump (LC-10AT vp, Shimadzu, Japan), an automatic injector (SPD-10AF, Shimadzu, Japan), a UV-VIS detector (SPD-10A vp, Shimadzu, Japan), a degasser (ERC-3415, Japan), and an Apollo C18 5 *μ* column (4.6 × 250 mm, Alltech Associates, Inc. USA) maintained at ambient temperature. The mobile phase consisted of acetonitrile (A) and 0.1% aqueous phosphoric acid (B) using a gradient elution of 90% A at 0–6 min, 90–78% A at 6–12 min, 78–72% A at 12–18 min, 72–67% A at 18–22 min, 67–55% A at 22–24 min, 55–50% A at 24–30 min, 50–70 A at 30–35 min, and 70–90% A at 35–40 min. The flow rate was 1.0 mL/min, the detection wavelength was 259 nm, and the injection volume was 20 *μ*L.

For the assay of polyphenols, daidzin, daidzein, genistin, and genistein were individually dissolved in methanol and 6,7-dimethyloxyl coumarin (6,7-DMC, 50.0 *μ*g/mL) was spiked as the internal standard. FME was dissolved in deionized water and spiked with the internal standard for HPLC/DAD analysis. The peak area ratios of each standard to the internal standard versus concentration of each standard were fitted to make the calibration curves.

### 2.4. Antioxidant Activities of FME

#### 2.4.1. Measurement of Reducing Power

The reducing power of FME was determined using a previously described method [3]. Briefly, serial dilutions of FME (10–500 *μ*g/mL) were prepared in phosphate buffer containing ferrocyanide and then incubated. Trichloroacetic acid was then added and the mixture was centrifuged. The supernatant was then mixed with an equal volume of distilled water containing 1% ferric chloride and the absorbance was measured at 700 nm. Ascorbic acid and distilled water were used as positive and negative controls, respectively. The absorbance intensity served as a surrogate measurement of antioxidant activity of the extract as follows:

(1) reducing  power  (%)  =(Acontrol  at  700 nm−Asample  at  700 nmAcontrol  at  700 nm)×100.

#### 2.4.2. Metal Chelating Activity

The chelating of ferrous ions by FME was estimated by the ferrozine assay with slight modifications as reported previously [3]. Briefly, FME (5–500 *μ*g/mL) was added to a solution of FeCl_2_. After that, the reaction was initiated by the addition of ferrozine and the mixture was shaken vigorously. The absorbance of the solution was then measured spectrophotometrically at 562 nm with an ELISA reader (Biotek, USA). Methanol served as the negative control and EDTA was used as the positive control. The results are expressed as a percentage of inhibition of the formation of the ferrozine-Fe^2+^ complex and were calculated by the following equation:

(2) chelating  effect  (%)  =(Acontrol  at  562 nm−Asample  at  562 nmAcontrol  at  562 nm)×100.

#### 2.4.3. DPPH Radical Scavenging Activity

Reaction mixtures containing a methanolic solution of DPPH and serial dilutions of sample ranging from 0.05 to 5 *μ*g/mL were placed in a 96-well microplate at room temperature in the dark for 30 min. After incubation, the absorbance was read at 492 nm by an ELISA reader (Tecan, Austria). Ascorbic acid was used as a positive control. The capacity to scavenge the DPPH radical was determined by the following equation:

(3) scavenging  effect  (%)  =(Acontrol  at  517 nm−Asample  at  517 nmAcontrol  at  517 nm)×100.

#### 2.4.4. Superoxide Anion Scavenging Assay

Reaction mixtures comprised 120 *μ*M phenazine methosulfate (PMS), 936 *μ*M dihydronicotinamide-adenine dinucleotide (NADH), 200 *μ*M nitroblue tetrazolium (NBT), 0.1 M phosphate buffer (pH 7.4), and the sample solution (5–500 *μ*g/mL). The mixtures were reacted at room temperature for 5 min and absorbance was read at 570 nm by an ELISA reader (Tecan, Austria). Ascorbic acid was used as a positive control.

#### 2.4.5. Peroxide Scavenging Assay

The ability of FME to scavenge H_2_O_2_ was determined spectrophotometrically as previously described [3]. Briefly, a 20 mM solution of H_2_O_2_ was prepared in PBS (pH 7.4), added to various concentrations of FME that had been dissolved in methanol (5–500 *μ*g/mL), and then allowed to stand at room temperature in the dark for 10 min. The absorption was measured at 230 nm by an ELISA reader (Tecan, Austria). The H_2_O_2_ scavenging activity of FME was determined by the following equation:

(4) scavenging  effect  (%)  =(Acontrol  at  230 nm−Asample  at  230  nmAcontrol  at  230 nm)×100.

#### 2.4.6. Determination of Hydroxyl Radical Scavenging Activity

The ability of FME to scavenge hydroxyl radical was measured according to a previously reported method [3]. Briefly, FME was dissolved in methanol to reach concentrations of 5, 10, 50, 100, and 500 *μ*g/mL. The assay was performed by adding FME, KH_2_PO_4_-KOH buffer, deoxyribose, FeCl_3_, EDTA, H_2_O_2_, and ascorbic acid. TBA and TCA were added and the mixture was incubated at 100°C for 15 min. Then, butanol-pyridine solution was added to develop the pink chromogen and the mixture was centrifuged. Absorbance of the supernatant was then measured at 532 nm by a microplate reader (Biotek, USA). The hydroxyl radical scavenging activity of FME is reported as percentage inhibition of deoxyribose degradation as per the following equation:

(5) inhibition  (%)  =(Acontrol  at  532 nm−Asample  at  532 nmAcontrol  at  532 nm)×100.

#### 2.4.7. AAPH-Induced Hemolysis Assay

Blood was obtained from male Sprague-Dawley rats via cardiopuncture and placed in tubes containing EDTA. This animal study adhered to *The Guidebook for the Care and Use of Laboratory Animals* (published by The Chinese Society for Laboratory Animal Science, Taiwan) and was approved by the Institutional Animal Care and Use Committee, China Medical University (Protocol no. 99-94-N). The *in vitro* resistance of intact red blood cells to oxidation was evaluated with AAPH as described previously [19]. The erythrocytes were isolated by centrifugation at 3000 ×g for 10 min, washed four times with PBS, and then resuspended to the desired hematocrit level using the same buffer. In order to induce free radical chain oxidation in the erythrocytes, aqueous peroxyl radicals were generated by thermal decomposition of AAPH in oxygen. An erythrocyte suspension was incubated with PBS (control) or with FME (10–500 *μ*g/mL), followed by incubation with or without 300 mM AAPH in PBS. The reaction mixture was removed and centrifuged, and the absorbance of the supernatant was measured at 540 nm. Reference values were determined using the same volume of erythrocytes in a hypotonic buffer. The extent of hemolysis was calculated using the following formula:

(6) hemolysis  (%)=[(AsampleAcontrol)]×100.

### 2.5. Screening of Antiphotoaging Activity of FME—MMP Activity Assays

Enzyme activity assays were performed in tris buffer, NaCl, and CaCl_2_. Various concentrations of FME were tested for their ability to digest a synthetic fluorogenic substrate (a general MMP substrate). Each concentration of FME was incubated with substrate and fluorescence intensity was measured at 320 nm (excitation) and 450 nm (emission) with a fluorescence reader. The rate of collagenase inhibition was calculated by the following equation:

(7) $$\begin{array}{c} \text{inhibition}\;\;\left(\%\right)=\left(1-\frac{\left(C-D\right)}{\left(A-B\right)}\right)\times 100, \end{array}$$

where *A* indicates the absorbance with enzyme but without sample, *B* indicates the absorbance without enzyme and sample, *C* indicates the absorbance with enzyme and sample, and *D* indicates the absorbance without enzyme but with sample.

### 2.6. Measurement of Elastase Activity

The test for elastase inhibition was conducted using elastase derived from porcine pancreas. Elastase was dissolved in tris buffer solution and elastase substrate IV was dissolved in tris buffer solution. To measure elastase activity, tris buffer solution, elastase substrate IV solution, sample solution, and elastase solution were dispensed into each well of a 96-well plate and then preincubated for 20 min at room temperature. Elastase activity was quantified by measuring light absorbance at 405 nm using a microplate reader (Tecan, Grodig, Austria). Each assay was carried out in triplicate.

The rate of inhibition of elastase was calculated by the following equation:

(8) $$\begin{array}{c} \text{inhibition}\;\;\left(\%\right)=\left(1-\frac{\left(C-D\right)}{\left(A-B\right)}\right)\times 100, \end{array}$$

where *A* indicates the absorbance with enzyme but without sample, *B* indicates the absorbance without enzyme and sample, *C* indicates the absorbance with enzyme and sample, and *D* indicates the absorbance without enzyme but with sample.

### 2.7. Cell Culture

Human foreskin fibroblasts (Hs68) were cultured in DMEM supplemented with 10% fetal bovine serum, 100 U/mL penicillin, and 100 U/mL streptomycin in a humidified atmosphere of 5% CO_2_ at 37°C.

### 2.8. UV Irradiation

Cells in PBS were exposed to UVB at a dosage of 40 mJ/cm^2^. A CL-1000 M UV crosslinker (UVP, Upland, CA, USA) was used as the UVB source and delivered a UVB energy spectrum of 302 nm. The cells were then incubated for 24 h at 37°C in a humidified atmosphere of 5% CO_2_ in serum-free DMEM containing various concentrations of FME.

### 2.9. MTT Assay for Cell Viability

Fibroblasts were plated at a density of 10^4^ cells/well in a 96-well plate and then treated with various concentrations of FME dissolved in DMSO (<0.1%) for 24 h. Mitochondrial dehydrogenase activity, which can be used as an index of cell viability, was assessed using the MTT assay [20]. Viability was quantified by measuring absorbance at 570 nm using a microplate reader (Tecan, Grodig, Austria).

### 2.10. Fluorescence Assay of Intracellular ROS

The intracellular concentration of ROS in fibroblasts was assessed using DCFDA (dichlorodihydrofluorescein diacetate) fluorescence dye. In brief, the assay involves incubating cells with DCFDA, which is then converted to the highly fluorescent molecule dichlorofluorescein (DCF) upon cleavage of the acetate groups by intracellular esterases. Fluorescence generated by reaction with ROS is then readily visible under a fluorescence microscope. Hs68 cells were seeded in a 24-well plate at a density of 10^5^ cells/well for 24 h and then exposed to UVB irradiation. After that, various concentrations of FME (1, 5, 10, and 50 *μ*g/mL) that had been prepared in serum-free DMEM were added and then incubated at 37°C for 24 h. Cells that had been prepared in DMEM were incubated at 37°C for 30 min in the presence of 10 *μ*M DCFDA. After that, the DMEM was removed and the cells were washed twice with 0.5 mL PBS. The cells were then covered with 0.5 mL PBS. Images were observed under a fluorescence microscope (Leica DMIL, German), and the fluorescence (emission 488 nm, excitation 520 nm) was measured using a microplate reader (Thermo Electron Corporation, Vantaa, Finland).

### 2.11. Western Blotting Analysis

Western blotting was performed using whole cell lysates prepared from Hs68 cells at a density of 5 × 10^5^ cells. Cells were harvested and homogenized with lysis buffer as previously described [19]. All reactions were performed in triplicate. Protein concentration in the culture medium was measured using Bradford reagent (Bio-Rad, Hercules, CA, USA) with bovine serum albumin as the standard. Cell lysates containing equal amounts of total protein were separated by electrophoresis on SDS-polyacrylamide gel and then transferred to a PVDF membrane (Amersham Pharmacia Biotech Inc., NJ, USA). Nonspecific binding was blocked with nonfat milk in TBST. The membrane was incubated with goat polyclonal antibodies against MMP-1 (1 : 500) and type I procollagen (1 : 500), and mouse polyclonal antibodies against MMP-3 (1 : 500), MMP-9 (1 : 500), ERK (1 : 500), JNK (1 : 500), p38 (1 : 500), p-ERK (1 : 500), p-JNK (1 : 500) and p-p38 (1 : 500) (Santa Cruz Biotechnology, Inc., CA, USA). Anti-immunoglobulin G-horseradish peroxidase (Santa Cruz Biotechnology Inc.) was used as the secondary antibody. Immunoreactive proteins were detected with the ECL Western blotting detection system (Fujifilm, LAS-4000, Japan). Signal strengths were quantified using a densitometric program (Multi Gauge V2.2).

### 2.12. Zymography for MMP-9

Cell-free medium after UVB exposure was mixed with tris-glycine SDS sample buffer without reducing agent and electrophoresed. After electrophoresis, the gels were incubated with zymogram renaturing buffer (Invitrogen, USA). Zymogram developing buffer (Invitrogen, USA) was then added to the gel. The gels were stained with 0.05% Coomassie Blue G-250 and destained in 10% acetic acid and 40% methanol until proteinase bands were clearly visible in a blue background. The area of light translucent zones over the blue background was determined by a densitometric program (Multi Gauge V2.2) to estimate gelatinase activity. One has

(9) relative  fluorescence  (%)=(Acontrol−AsampleAcontrol)×100.

### 2.13. Statistical Analysis

Each experiment was performed in triplicate and all data are presented as mean ± SD. Significant differences between groups were analyzed by ANOVA followed by the Scheffe's test. A *P* value < 0.05 was considered to represent statistical significance.

## 3. Results

### 3.1. Extraction Yield of FME and Quantitation of Phenolic Content

**Figure 1:**
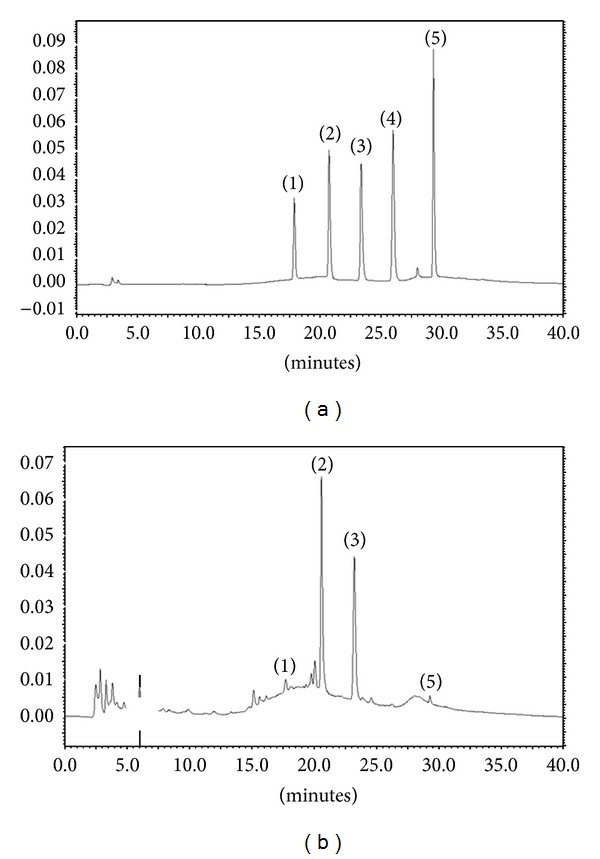
HPLC chromatograms of constituents in FME. (a): Standards, (b): FME. (1) Daidzin, (2) genistin, (3) 6,7-DMC (internal standard), (4) daidzein, and (5) genistein.

The extraction yield of FME was 18.2%. The total phenolic content in the extract, as determined by the Folin-Ciocalteu method [19], was 215 *μ*g GAE/mg. HPLC chromatograms of FME and its constituents are shown in Figure 1. The isoflavonoids in FME and the internal standard were well resolved within 40 min by gradient elution. FME in this study contained 0.31 ± 0.03 mg of daidzin per mg FME, 2.20 ± 0.18 mg of genistin per mg of FME, and 0.12 ± 0.02 mg of genistein per mg of FME. No daidzein was detected.

### 3.2. Measurement of Reducing Power

**Figure 2:**
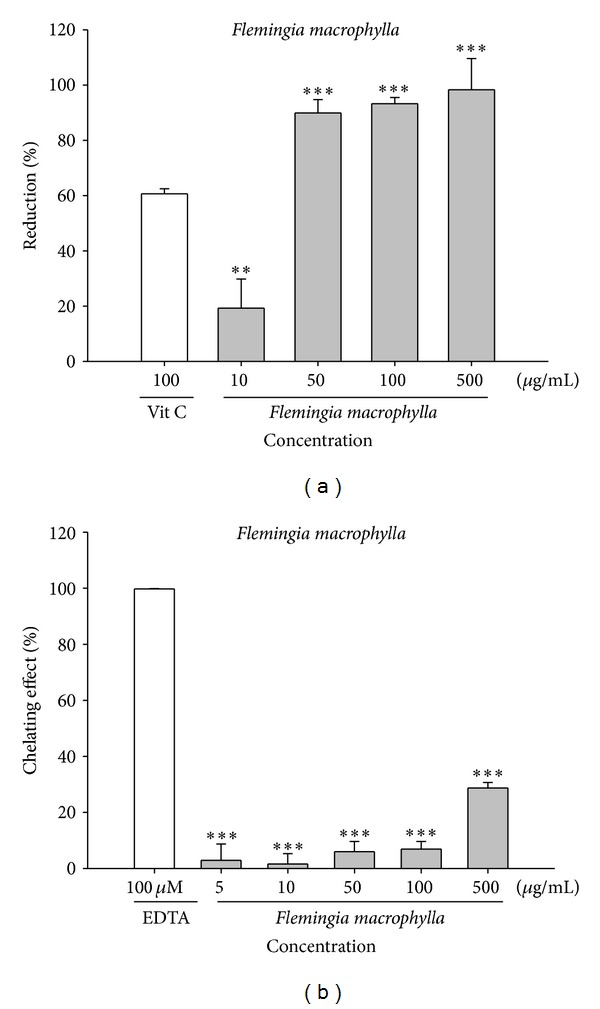
The reducing capacity (a) and ferrous chelation activity (b) of FME. Ascorbic acid and EDTA were applied as positive control, respectively. (*n* = 4; significant difference versus control (without extract): **P < 0.01; ***P < 0.001; significant difference versus ascorbic acid).

The reducing capacity of FME was 19.2 ± 10.5% at 10 *μ*g/mL, 89.9 ± 4.7% at 50 *μ*g/mL, 93.3 ± 2.2% at 100 *μ*g/mL, and 98.3 ± 11.2% at 500 *μ*g/mL (Figure 2(a)). The reducing capacity of the positive control (100 *μ*g/mL ascorbic acid) was 60.2 ± 2.1%. The IC_50_ value of FME was 27.4 *μ*g/mL. The results clearly indicate that the reducing capacity of FME is superior to that of ascorbic acid.

### 3.3. Metal Chelating Activity

Figure 2(b) shows the metal chelating activities of FME and the positive control EDTA (100 *μ*M). The activities of various concentrations of FME (range: 5 to 500 *μ*g/mL) ranged from 2.9 ± 5.8% to 28.7 ± 1.9% and that of EDTA was 100.0 ± 0.3%. The results show that the metal chelating ability of FME is inferior to that of EDTA.

### 3.4. DPPH Radical Scavenging Activity of FME

**Figure 3:**
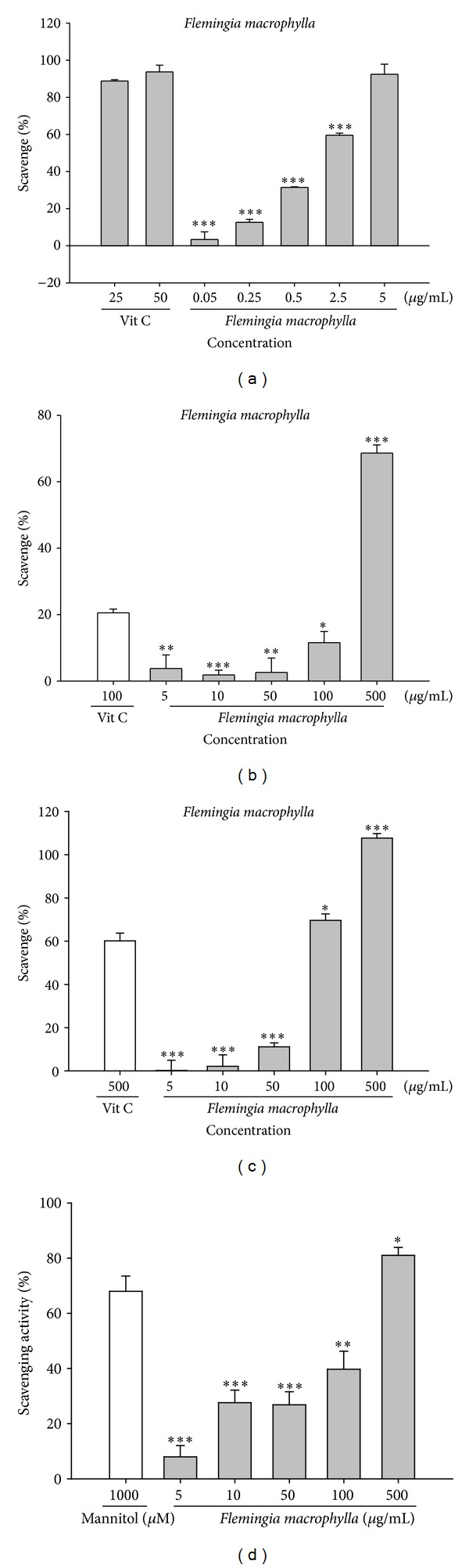
The free radical scavenging activities of FME. (a) DPPH radical scavenging activity; (b) superoxide scavenging activity; (c) peroxide radical scavenging activity; and (d) hydroxyl radical scavenging activity of FME (*n* = 4; ***P < 0.001).

DPPH is commonly used as a reagent to evaluate free radical scavenging activities of antioxidants.  Figure 3(a) shows the free radical scavenging activity of FME (0.05–5 *μ*g/mL) and ascorbic acid (25 and 50 *μ*g/mL). We found that FME scavenged DPPH radicals in a dose-dependent manner. The results of the experiment show that FME exhibited a scavenging activity of 92.9 ± 5.4% at low dosage (5 *μ*g/mL), which was similar to the activity of ascorbic acid at a concentration of 50 *μ*g/mL. At lower doses (2.5 *μ*g/mL) the radical scavenging activity was still approximately 60% and the IC_50_ of FME was 2.1 *μ*g/mL. This finding indicates that FME is a potent antioxidant.

### 3.5. Superoxide Anion Radical Scavenging Activity of FME

In this assay, PMS was mixed with *β*-NADH to produce superoxide anion and NBT was added to facilitate the conversion of tetrazolium to diformazan, which absorbs light at 560 nm. As seen in Figure 3(b), the scavenging activity of 100 *μ*g/mL ascorbic acid (positive control) was 20.1 ± 1.4% and that of FME ranged from 3.7 ± 4.1% at a concentration of 5 *μ*g/mL to 68.6 ± 2.4% at a concentration of 500 *μ*g/mL. The IC_50_ of FME was 366.8 *μ*g/mL.

### 3.6. Peroxide Scavenging Assay

Peroxide is the primary product of oxidation produced in oxidative processes. This reactive oxygen species penetrates the cell membrane and reacts with Fe^2+^ or Cu^2+^ to form hydroxyl radicals. In addition, hydrogen peroxide has been shown to inactive the thiol group of some enzymes [21]. The peroxide scavenging activities of FME and ascorbic acid, the positive control, are shown in Figure 3(c). The peroxide radical scavenging activities of various concentrations of FME (5 to 500 *μ*g/mL) ranged from 0.2 ± 4.7% to 107.7 ± 2.1%, while the peroxide radical scavenging activity of ascorbic acid (500 *μ*g/mL) was 60%. The IC_50_ of FME was 178.9 *μ*g/mL. The radical scavenging activity of FME at 100 *μ*g/mL was similar to that of ascorbic acid at 500 *μ*g/mL.

### 3.7. Determination of Hydroxyl Radical Scavenging Activity

Hydrogen peroxide generates hydroxyl radicals via the Fenton reaction [22]. Hydroxyl radicals oxidize 2-deoxyribose, which then reacts with thiobarbituric acid (TBA) to form a chemophore. The hydroxyl radical scavenging activities of FME and the positive control mannitol are shown in Figure 3(d). The hydroxyl radical scavenging activities of various concentrations of FME (5 to 500 *μ*g/mL) ranged from 7.9 ± 4.1% to 81.0 ± 2.9% while that of mannitol (1,000 *μ*M) was 68.0 ± 5.5%. The IC_50_ of FME was 230.9 *μ*g/mL.

### 3.8. The Inhibitory Effect of FME on AAPH-Induced Erythrocyte Hemolysis

**Figure 4:**
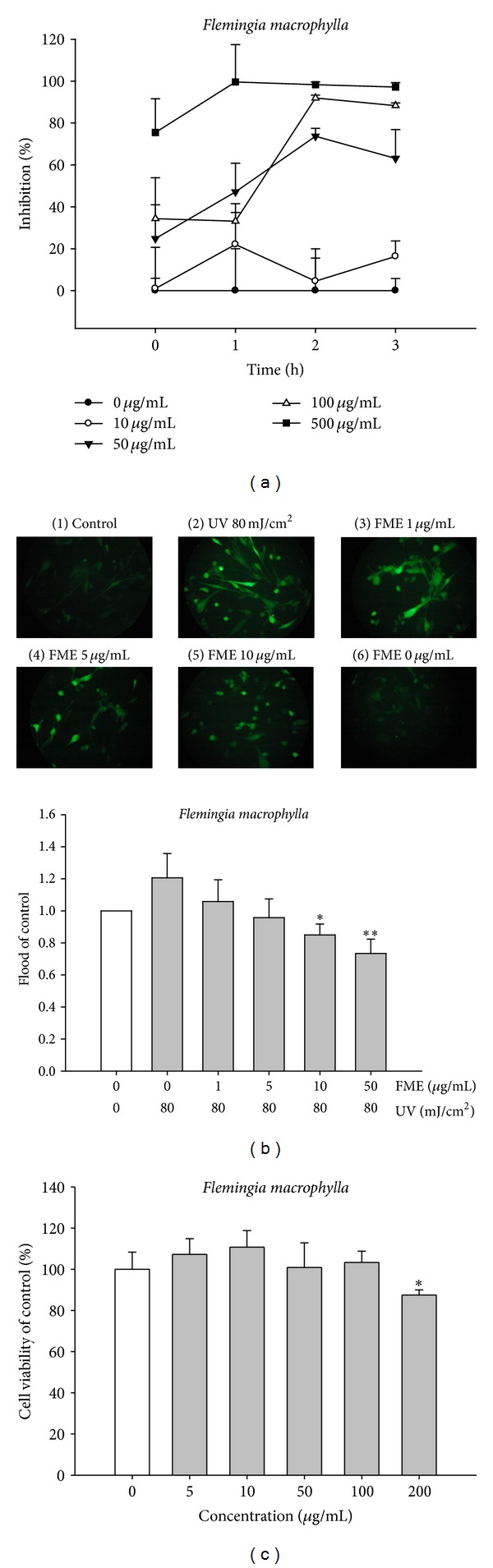
The antioxidant activities of FME in cell and cell viability. (a) AAPH-induced erythrocyte hemolysis. (b) Effect of FME on UVB-induced ROS generation in fibroblasts. (c) The cell viability of FME in fibroblasts.

AAPH-induced hemolysis is a commonly used model for studying biomembrane damage [23]. In this study, we found that treatment of erythrocytes with AAPH resulted in hemolysis. However, we found that the degree of AAPH-induced hemolysis was time dependent (Figure 4(a)). Erythrocytes incubated with FME at 50 *μ*g/mL for 2 h exhibited a significant reduction in the rate of AAPH-induced hemolysis (Figure 4(a)). The protective effect of FME on AAPH-induced hemolysis was dose dependent. These results indicate that FME exhibits potent AAPH radical scavenging activity.

### 3.9. Fluorescence Assay of Intracellular ROS

The antioxidant activity of FME in UVB-exposed fibroblasts was determined by measuring the intracellular ROS generation using the DCFDA-ROS detection assay. In our preliminary study, the fluorescence intensity increased in proportion to the UVB dose (data not shown). As shown in Figure 4(b), the levels of ROS were markedly higher in UVB-exposed fibroblasts than in control cells; however, this induction of ROS generation was attenuated in a dose-dependent manner by pretreating UVB-exposed fibroblasts with various concentrations of FME (1–50 *μ*g/mL). Taken together, the results mentioned above indicate that FME protects fibroblasts from UV-induced oxidative stress by scavenging various free radicals.

### 3.10. Effect of FME on Cell Viability

To test the *toxicity* of FME in fibroblasts, cells were treated with various concentrations of FME and cell viability was measured using the MTT assay. As shown in Figure 4(c), the cell viabilities of various concentrations of FME were similar to that of the control group. The results indicated that FME did not affect the viability of the tested cell line (up to 200 *μ*g/mL) and that the viability of cells exposed to 200 *μ*g/mL FME (in terms of percentage) was 87.5 ± 2.3% (Figure 4(c)).

### 3.11. Fluorometric Analysis of the Inhibitory Effect of FME on Bacterial Collagenase-1 Activity

**Figure 5:**
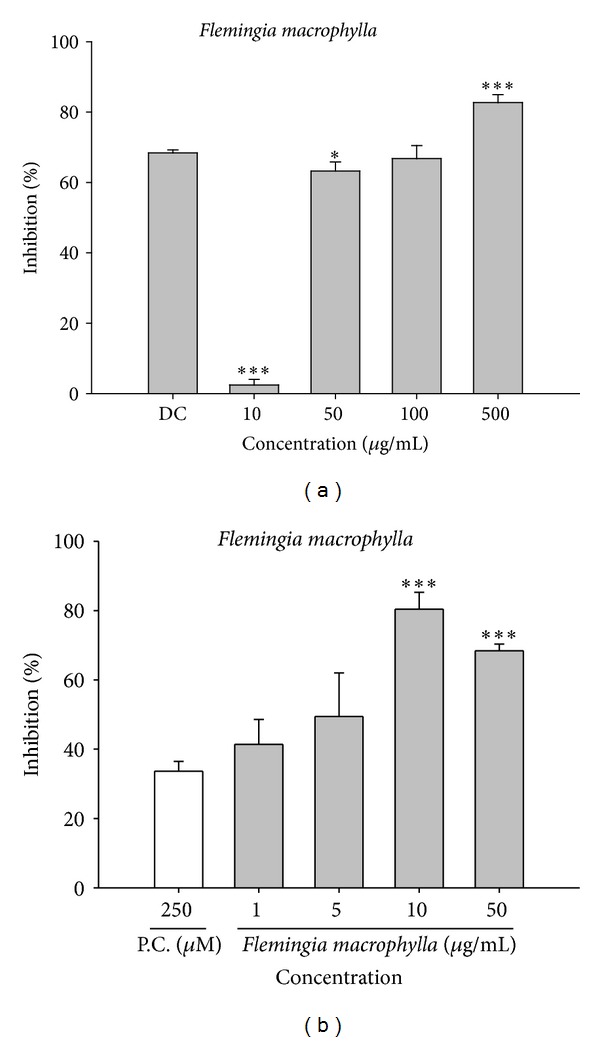
The assay of FME on collagenase and elastase activities. (a) The inhibition rate (%) of FME on bacterial collagenase activity using fluorometric assay. (b) The inhibition rate of FME on porcine elastase. DC: doxycycline; PG: propylene glycol; P.C.: positive control, elastase inhibitor I.

Fluorescence-conjugated substrate was incubated with bacterial collagenase-1 in the presence of different concentrations of FME or doxycycline hyclate (positive control). As shown in Figure 5(a), treatment with 50 *μ*g/mL of FME resulted in a 63.3 ± 2.6% decrease in enzyme activity and treatment with 500 *μ*g/mL resulted in an 82.7 ± 2.2% decrease in collagenase-1 activity, relative to the control, indicating that FME significantly inhibits the activity of bacterial collagenase-1.

### 3.12. The Effect of FME on Elastase Activity

We found that FME inhibited elastase activity (range: 41.5 ± 7.0% at 1 *μ*g/mL to 80.0 ± 4.4% at 10 *μ*g/mL) and that the inhibitory effect elicited by FME at 10 *μ*g/mL (80.0 ± 4.4%) was more significant than that elicited by elastase inhibitor I (positive control) at 250 *μ*M (32.6 ± 3.0%) (Figure 5(b)).

### 3.13. FME Upregulates Type I Procollagen Production

**Figure 6:**
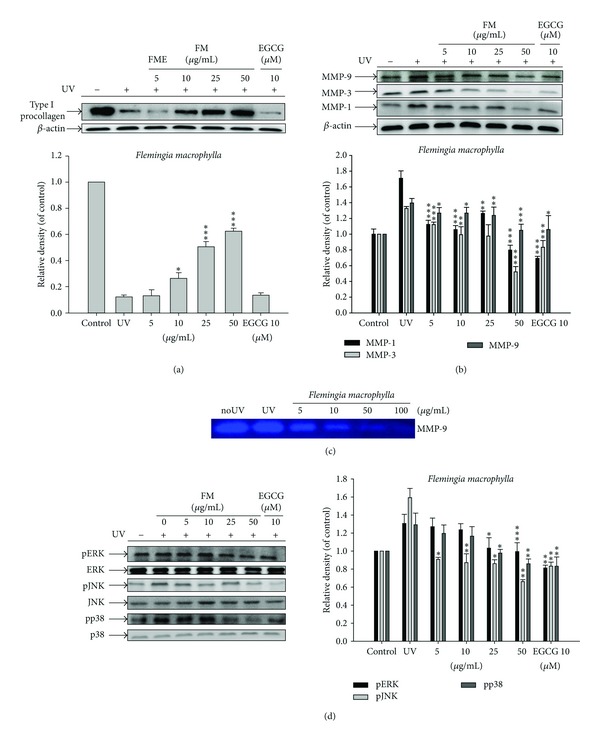
Effect of FME on the UV-induced (a) type I procollagen expression in human fibroblasts; (b) MMP-1, MMP-3, and MMP-9 expression in human fibroblasts; (c) MMP-9 by gelatin zymography in the culture medium of human fibroblasts; and (d) MAP kinases expression in human fibroblasts.

Fibroblasts were treated with FME (5–50 *μ*g/mL) for 24 h after exposure to UVB (80 mJ/cm^2^) and the levels of type I procollagen were measured using Western blot. EGCG was used as the positive control. FME treatment (≥10 *μ*g/mL) resulted in a significant increase in the production of type I procollagen relative to that of control cells (Figure 6(a)).

### 3.14. FME Inhibited the Protein Expression of MMPs

In Hs68 cells, UVB treatment caused a 1.7-fold increase in MMP-1 expression, a 1.3-fold increase in MMP-3 expression, and a 1.4-fold increase in MMP-9 expression relative to control levels. FME treatment (5–50 *μ*g/mL), however, suppressed the UVB-induced upregulation of MMPs in a dose-dependent manner (Figure 6(b)). FME at concentrations of 5 *μ*g/mL and above led to a significant decrease in protein expression of MMP-1 and MMP-3 (Figure 6(b)).

### 3.15. Effect of FME on UVB-Induced MMP-9 Secretion

MMP-9 is a UVB-inducible MMP that plays an important role in photoaging. We found that the activity of MMP-9 in fibroblasts exposed to UVB irradiation was higher than that in control cells. FME inhibited MMP-9 activity in a dose-dependent manner (5–100 *μ*g/mL) (Figure 6(c)).

### 3.16. Effect of FME on MAP Kinase Expression

UVB-induced MAP kinase phosphorylation causes the secretion of collagen-degrading proteins MMP-1, -3, and -9, resulting in photoaging. UVB would not influence MAP kinase expression, but it would significantly activate the phosphorylation of MAP kinases in human skin fibroblasts. In this study, we found that UVB irradiation of Hs68 cells resulted in a 1.3-fold increase in p-ERK expression, a 1.6-fold increase in p-JNK expression, and a 1.3-fold increase in phosphorylated p38 relative to control levels (Figure 6(d)). FME treatment (5–50 *μ*g/mL), however, significantly reversed the UVB-induced overexpression of those MAP kinases. The levels of p-ERK were suppressed to basal levels at 25 *μ*g/mL FME and the levels of p-JNK were reduced to basal levels at 5 *μ*g/mL FME. The levels of phosphorylated p38, however, were reduced to basal levels at 25 *μ*g/mL FME (Figure 6(d)).

## 4. Discussion

In this study, we found that* Flemingia macrophylla *extract (FME) had high reducing capacity, was a potent free radical scavenger, ameliorated UVB-induced ROS generation *in vivo*, suppressed UVB-induced overexpression of MMPs, inhibited elastase activity, protected against UVB-induced overexpression of type I procollagen, and inhibited UVB-induced phosphorylation of MAP kinases. Based on our results, FME appears to be a potent antioxidant that can protect against photoaging-related skin damage.

Crude extracts of plant materials that are rich in phenolic or isoflavonoid content are of interest in the health food and cosmetic industries because of their antioxidant and tumor preventing activities. Assays that measure total phenolic and flavonoid content are fast and convenient methods for screening polyphenol-rich materials. Our results indicate that FME is a potent antioxidant and that it inhibits photoinduced expression and secretion of MMPs. The results of the HPLC-DAD analysis in this study suggest that the major isoflavonoids in FME are daidzin, genistin, and genistein. It has been reported that these three isoflavonoids and their derivatives are potent antioxidants and UV damage preventers [16, 17, 24]. Therefore, these isoflavonoids and their related compounds may contribute to the antioxidant and photodamage preventing properties of FME.

Reactive oxygen species are known to play a role in UV-induced skin damage and aging. High levels of ROS, including superoxide anion, hydrogen peroxide, and hydroxyl radical, can induce various human diseases. Results of the antioxidant assays in this study showed that FME at 500 *μ*g/mL scavenged 107.7 ± 2.1% of the available H_2_O_2_ species, 68.6 ± 2.4% of the available superoxide species, and 81.0 ± 2.9% of the available hydroxyl radical species. In addition, FME demonstrated greater reducing power than ascorbic acid (100 *μ*g/mL). These results indicate that FME is a powerful antioxidant.

Plant polyphenols are known to inhibit lipid peroxidation by quenching lipid peroxyl radicals and by reducing or chelating iron in cells [25]. We found that FME protected against AAPH-induced hemolysis in a dose-dependent and time-dependent manner and attenuated UV-induced generation of intracellular ROS. The results are consistent with those obtained from the analyses of reducing power, DPPH scavenging activity, and free radical scavenging activity. The antioxidant activity of FME may be due to its redox properties, which allow it to act as a reducing agent, hydrogen donor, and singlet oxygen quencher. UV exposure can lead to the depletion of endogenous antioxidants resulting in ROS-induced aging and DNA damage, especially in skin. FME with its UVB-induced ROS quenching effect might, therefore, be an effective antiphotoaging agent.

UV irradiation results in photoaging and induces ROS formation, which triggers complex signaling pathways including MMPs overexpression and degradation of ECM in connective tissues [2, 26]. ROS interacts with proteins, lipids, and DNA, resulting in photoaging, skin damage, and cancer. MMP-1 is mainly responsible for the degradation of dermal collagen during the ageing process whereas MMP-9 is known to degrade type IV collagen, which is an important component of the basement membrane of the skin dermal epidermal junction. In addition to collagen, elastin is an important ECM protein in dermis and the degradation of elastin has been shown to cause line and wrinkle formation in the skin. Tsuji et al. found that damage to the elastic fiber network in hairless mouse skin was responsible for UVB-exposed skin wrinkling [27]. Agents that inhibit collagenase and elastase activity are ideal candidates for the prevention or treatment of photoaging. We found that FME exhibited good elastase inhibiting activity, inhibited MMP-1, -3, and -9 expression, and elevated type I procollagen production. Studies have shown that genistin inhibits UV-induced DNA damage in human melanoma cells and that isoflavones in soybean extract inhibit UVB-induced ROS, JNK activation, cyclooxygenase-2 activation, and proliferation of cell nuclear antigen in keratinocytes [28, 29]. FME, which is rich in the isoflavones genistin, genistein, and daidzin that are found in soybean and other natural products, might contribute to the antiphotoaging activity of FME.

MAP kinases are the upstream regulators of MMP proteins [30–32]. In this study, FME significantly suppressed the overexpression of UVB-induced p-ERK, p-JNK, and phosphorylated p38. It has been shown that UV irradiation induces p38 activation [32]. Furthermore, it has been reported that UVB irradiation induces ERK, JNK, and p38 activation via production of ROS [33–35] and that the activation of these kinases correlated with skin death in response to UVB [36]. In our study, FME exhibited potent antioxidant activity, inhibited the expression of MAP kinases and MMPs, and restored type I procollagen to basal levels. Those findings indicate that FME is a potential agent for the treatment or prevention of photodamaged skin. FME hampers the activation of MAP kinases and, therefore, may downregulate the downstream proteins c-Fos and c-Jun, as well as the transcription factor AP-1 and other proto-oncogenes involved in photoaging. The effects of FME on transcription of c-Fos, c-Jun, and AP-1 require further study.

## 5. Conclusions

**Figure 7:**
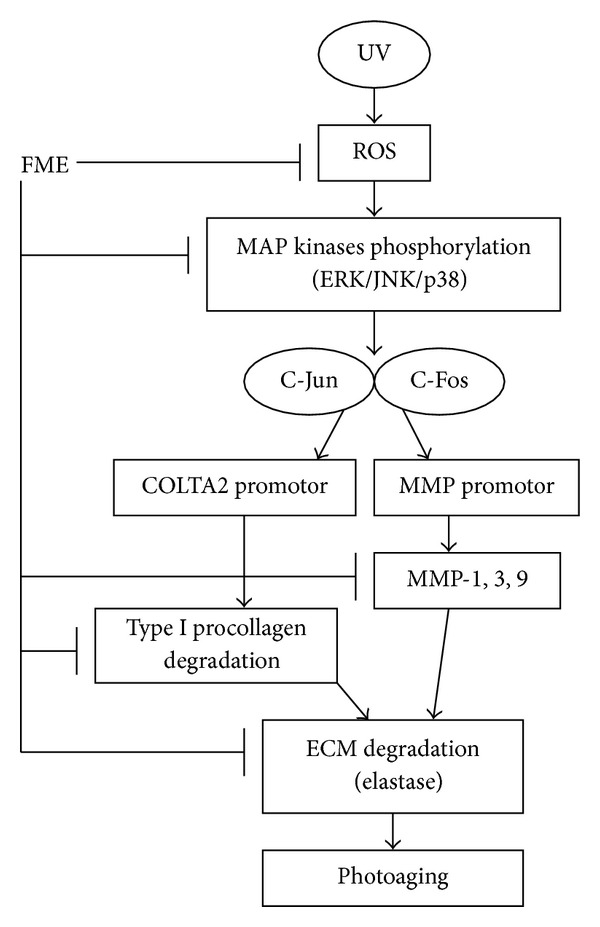
The scheme of FME on antioxidant and antiphotoaging.

We found that FME inhibited UVB-induced ROS generation and UVB-induced overexpression of phosphorylated p38, JNK, and ERK and attenuated the overexpression of MMP-1, -3, and -9, thereby elevating type I procollagen synthesis (Figure 7). FME, therefore, could be an ideal antiaging and antiphotoaging agent.
